# Set-shifting and selective attentional impairment in alcoholism and its relation with drinking variables

**DOI:** 10.4103/0019-5545.31619

**Published:** 2006

**Authors:** Nirmal Saraswat, Sanjeev Ranjan, Daya Ram

**Affiliations:** * PhD Scholar, Department of Clinical Psychology, National Institute of Mental Health and Neurosciences, Bangalore, India; ** Senior Resident, Department of Psychiatry, NIMHANS; *** Professor, Central Institute of Psychiatry, Ranchi, India

**Keywords:** Frontal lobe, alcohol, executive functions, set-shifting, selective attention

## Abstract

**Background:**

Individuals with chronic alcoholism show impairments in visual scanning, set-shifting and response inhibition abilities.

**Aim:**

To study the relationship between performance on tests of set-shifting and selective attention, and alcohol intake variables (duration of dependence, amount of alcohol intake, and duration of abstinence during the past year).

**Methods:**

In this cross-sectional, controlled study, inpatients from a tertiary care centre were selected. Thirty patients with alcohol dependence and 15 age-, sex- and education-matched normal controls were administered the Trail Making Test (TMT) and Stroop test to assess visual scanning, set-shifting and response inhibition abilities. The data were analysed using the χ^2^ test, *t* test and ANOVA with post-hoc analysis.

**Results:**

The patient group performed poorly on all measures of the tests. The duration of dependence and the amount of alcohol intake (during the past 1 year) were not found to significantly affect the performance on the 2 tests. The duration of abstinence during the past 1 year was significantly related to performance on the Stroop test with patients having a longer duration of abstinence showing lesser impairment.

**Conclusion:**

Patients with a fewer number of days of alcohol intake during the past 1 year show relatively better visual scanning, set-shifting and response inhibition abilities.

## INTRODUCTION

Individuals with chronic alcohol abuse show impairments in several cognitive functions. Impairments in memory, abstracting ability, motor efficiency, visuo spatial integration and learning have been reported in individuals who have a history of chronic alcohol abuse.^1^ Based on different findings relating to different areas of the brain, several hypotheses have been proposed including the right brain deficit hypothesis, the diffuse brain deficit hypothesis and the anterior brain deficit hypothesis.^2^–^5^ The current literature emphasizes a primary hypothesis concerning the ‘anterior brain deficit’ in alcoholics. Considerable research has focused on the nature and extent of frontal lobe involvement in alcoholism. Individuals with alcoholism are reported to be impaired on tests of functions related to the frontal lobes.^5^–^7^ Frontal lobe deficits tend to interfere with relapse prevention strategies directed at the rehabilitation of alcohol-dependent patients after the detoxification phase is over.^8^ It is not known how alcohol consumption variables affect frontal lobe functions. The limited effort made in this direction has produced inconclusive results.^9^–^11^ A clearer understanding of this relationship is required for designing appropriate intervention programmes. This was the driving force behind the present study.

The study attempts to examine frontal lobe functions and their association with alcohol consumption variables, i.e. duration of dependence, amount of intake and pattern of consumption in patients with alcohol dependence.

## METHODS

This cross-sectional assessment study was conducted between August 2001 and March 2002. The sample for the study consisted of 30 patients and 15 normal controls. The selected patients met the following inclusion criteria:

- Age 18–50 years
- Diagnosed as alcohol dependent according to the diagnostic criteria of ICD-10 for research.^12^
- Abstinence from alcohol for at least 3 weeks
- Not taking any psychotropic drug for one week
- Right-handed
- No history of head injury, clinically apparent neurological/medical problems, previous or present psychiatric illness including dementia, mental retardation, colour blindness, and any other drug abuse except tobacco.

Consecutively admitted patients to the hospital, who met the inclusion criteria, were recruited into the study. Since the hospital admits only male patients, all the participants in the study were males. The control group consisted of 15 normal subjects, who were matched in terms of age, education, socioeconomic status and handedness with every alternate patient from the experimental group and who had no history of alcohol abuse. The control subjects were volunteers selected from the local community. A General Health Questionnaire score of more than 2 was considered as the cut-off point for the control group. The criteria for exclusion of control subjects were similar to those for the experimental group. Informed consent was taken from all the participants after a detailed discussion about the nature of investigations. No financial benefits were provided to the participants. The study was reviewed and approved by the ethics committee of the institute.

### Tools

#### Sociodemographic and clinical data sheet

A semi-structured format especially designed for this study was used for recording the sociodemographic details of the patients, duration of alcohol consumption, age at onset, frequency, amount, pattern of use, last intake, history of abstinence, past illness and family history.

#### Trail making test (TMT)

This is a test of speed for visual scanning, attention, cognitive flexibility, set-shifting and motor function.^13^ This test is divided into two tasks. In task A the subject is asked to connect 25 consecutively numbered circles with a pencil, and in task B the subject is required to connect a series of numbered and lettered circles, alternating between the two sequences. Three scores are obtained: time (seconds) taken to complete task A, time (seconds) taken to complete task B, and the time difference (task B minus task A) between the two. This test is considered as a sensitive means to detect prefrontal cortical dysfunction.^14^

#### Stroop test

The Stroop test measures the ease with which a person can shift his or her perceptual set to conform to the changing demands and suppress a habitual response in favour of an unusual one.^13^ This measure of cognitive flexibility and selective attention was originally developed by John Ridley Stroop.^15^ In the present study, the Victoria version was used.^16^ It consists of three cards, each containing six rows of four items. In part D, the subject has to name as quickly as possible the colour of 24 dots printed in blue, green, red or yellow. Each colour is used six times, and four colours are arranged in a pseudorandom order within the array, each colour appearing once in each row. Part W is similar to part D, except that the dots are replaced by common words, printed in lower case. The subject is required to name the colours in which the stimuli are printed, and to disregard their verbal content. In part C, the coloured stimuli are the colour names 'blue, green, red and yellow' printed in lower case so that the print colour does not correspond to the colour name. The anterior cingulate is found to mediate processes involved in Stroop interference.^17^

#### General Health Questionnaire-5 (GHQ-5)

This is the shortest standardized version of the General Health Questionnaire designed by Goldberg in 1972.^18^ Those scoring 2 or above are considered as psychiatrically ill.

#### Handedness scale

In this 6-item scale the subject is asked to do some everyday activities such as writing a letter. Depending on the hand he uses for all the 6 items; he is assigned to that particular hand preference.^19^

All the subjects included in the present study were interviewed and then assessed by a single interviewer with the help of a semi-structured clinical data sheet. Details of the history of present illness, past illness, physical and mental status examination were noted. Detailed physical examination (including neurological examination) as well as various investigations were performed by the treating team if needed. GHQ-5 was administered on normal controls and only those who scored less than 2 were included in the study. Each group was assessed on the handedness scale and only right-handed persons were included in the sample to avoid the confounding effect of handedness. After initial screening, all the 45 subjects were administered TMT and the Stroop test as per the standard procedures.

To investigate the effect of amount of intake, the average amount of intake during the past 1 year was calculated. To see the effect of the abstinence period, the alcohol-dependence group was split into 3 subgroups based on the duration of abstinence during the past 1 year. Group I consisted of those subjects who were abstinent for less than 3 months; group II consisted of the subjects who were abstinent for 3–6 months, and group III consisted of the subjects who were abstinent for more than 6 months during the past 1 year.

### Statistical analysis

Statistical calculations were done using the computerized version of the statistical package for social sciences version 10.0. Group differences for clinical and demographic data were calculated using the χ^2^ test and *t* test. The *t* test was used to compute the group differences on the TMT and Stroop test scores. To compare the groups on the basis of duration of abstinence, ANOVA was carried out with post-hoc analysis using the Bonferroni correction method. Pearson correlation coefficient was computed to find out the relationship between the amount of intake, and duration of dependence with the TMT and Stroop test scores.

## Results

The groups were comparable regarding education, occupation, socioeconomic status, marital status, residence and religion, as none of the group differences were found to be statistically significant (Table 1). The mean age at onset of alcoholism was 17.86±4.23 years (range 10–34 years). On an average, patients had 10.94±3.31 years of alcohol dependence. The mean amount of alcohol consumption was 839.34±28.97 g/day. At the time of assessment, alcohol-dependent patients were maintaining abstinence for an average of 25.31 days.

**Table 1:** Characteristics of the study participants

	Groups				
	
Variables	Study *n* (%)	Control *n* (%)	p	df	95% CI
*Age* (years)					
mean (SD)	34.5±7.23	31.93±6.37	0.251*	43	−1.88-7.01
*Education*					
Up to matric	14 (46.7)	7 (46.7)	0.829†	2	
Secondary	6 (20.0)	2 (13.3)			
Graduate	10 (33.3)	6 (40.0)			
*Occupation*					
Unskilled	6 (20.0)	3 (20.0)	0.837†	2	
Skilled	20 (66.7)	9 (60.0)			
Unemployed	4 (13.3)	3 (20.0)			
*Socioeconomic status*					
High	14 (46.7)	9 (60.0)	0.543†	2
Middle	15 (50.0)	5 (33.3)			
Low	1 (3.3)	1 (6.7)			
*Marital status*				
Single	8 (26.7)	6 (40.0)	0.362†	1	
Married	22 (73.3)	9 (60.0)			
*Residence*					
Urban	22 (73.3)	11 (73.3)	1.00†	1	
Rural	8 (26.7)	4 (26.7)			
*Religion*					
Hindu	19 (63.3)	11 (43.3)	0.502†	1	
Christian	11 (36.7)	4 (26.7)			

* *t* test;

† χ2

The patient group performed poorly compared to the control group on all measures of the TMT and Stroop test (p<0.001) (Table 2). Correlation of the TMT and Stroop test scores of the patient group with the duration of dependence and amount consumed were not found to be statistically significant (Table 3).

**Table 2:** Comparison of the test scores of alcohol-dependent patients and controls

	Groups				
	
Variables	Study Mean±SD	Control Mean±SD	df	p	95% CI
TMT* part A	61.4 ± 24.51	33.9 ± 10.29	43	<0.001	14.09-40.84
TMT part B	158.3 ± 72.84	77.53 ± 25.34	43	<0.001	41.52-120.01
TMT part B-part A	96.9 ± 58.99	43.6 ± 18.76	43	<0.001	21.66-84.94
Stroop test part D	24.27 ± 11.35	16.00 ± 5.05	43	0.017	1.44-13.89
Stroop test part W	31.43 ± 14.83	18.07 ± 3.79	43	<0.001	5.48-21.26
Stroop test part C	44.57 ± 17.25	28.27 ± 7.99	43	<0.001	6.71-25.69

* TMT: Trail making test

**Table 3:** Correlations (r) of test scores with duration of dependence and amount of alcohol intake

	TMT* part A	TMT part B	TMT part B	Stroop test part D	Stroop test part W	Stroop test part C
Duration of dependence	0.29	0.26	0.21	0.1	0.11	0.08
Amount of intake	−0.1	0.09	0.15	−0.06	0.02	0.13

* TMT: Trail making test

The alcohol-dependent group was split into 3 sub-groups based on the total duration of abstinence during the 1 year. The first group consisted of subjects abstinent for less than 3 months; the second group consisted of subjects abstinent for 3–6 months and the third group consisted of subjects who were abstinent for more than 6 months. The three groups did not differ with respect to age, education and occupational status. The ANOVA calculated for the 3 groups revealed significantly better performance on card-C of the Stroop test by the group which was abstinent for more than 6 months during the past 1 year (Table 4).

**Table 4:** Comparison of scores of the tests for patients with different durations of abstinence

	Groups				
	
Variables	Abstinence (<3 months) *n*=11	Abstinence (3-6 months) *n*=11	Abstinence (>6 months) *n*=8	df	p
TMT* part A	67.45 ± 32.77	57.55 ± 15.02	58.38 ± 23.27	2, 27	0.603
TMT part B	174.45 ± 88.68	170.64 ± 65.39	119.13 ± 47.28	2, 27	0.210
TMT part B-part A	107.00 ± 61.51	113.09 ± 62.89	60.75 ± 36.01	2, 27	0.124
Stroop test part D	29.91 ± 13.48	22.18 ± 6.82	19.38 ± 11.12	2, 27	0.099
Stroop test part W	38.18 ± 18.26	30.45 ± 12.20	23.50 ± 8.82	2, 27	0.096
Stroop test part C	56.91 ± 16.43	38.91 ± 11.98	35.00 ± 15.73	2, 27	0.005

* TMT: Trail making test

## DISCUSSION

This study has some key findings. The patient group performed poorer than the control group on the Stroop test and TMT. However, these performances were not significantly related to the amount and duration of alcohol intake. Additionally, patients who were abstinent for more than 6 months during the past 1 year performed better than those with a lesser duration of abstinence during the past 1 year on the Stroop test.

The alcohol-dependent group required a significantly longer time to complete both part A and part B of the TMT. Significantly poor performance on TMT part A suggests impaired visual scanning and psychomotor speed, whereas significant group differences between the alcohol-dependent group and controls on TMT part B and part B minus part A indicate impaired cognitive flexibility and set-shifting in addition to impaired visual scanning and psychomotor speed. Alcohol-dependent patients also performed poorly on the Stroop test, which suggests impaired ability to suppress a habitual response, to focus attention selectively and to shift a perceptual set to conform to changing demands. These cognitive abilities are included under the executive functions carried out by the prefrontal cortex. Thus, impaired performance on these tests highlights the involvement of the prefrontal cortex. Our findings are in line with the findings of earlier studies.^1^,^3^ Poor performances by alcohol-dependent patients on tests of executive functions have been demonstrated by researchers using other tests as well as neurophysiological measures such as positron emission tomograms and evoked response potentials.^1^,^20^ Alcohol-dependent patients have decreased frontal perfusion as revealed by blood flow measured by ^99^mTc-bicisate SPECT.^21^ Thus, poorer executive functions in this population are not just an epiphenomenon of alcohol use but reflect the underlying pathophysiological substrate which in turn is the result of long-term alcohol use. Deficits in executive functions have important implications for the management of these patients. Poorer executive abilities correlate with the degree of denial of the dependence problem and hence interfere with the engagement of patients in therapeutic interventions.^22^,^23^

The significant relationship between the duration of abstinence (during the past year) and performance on the Stroop test part C is the other positive finding of this study. This relationship reached significance only with part C of the Stroop test probably because it is relatively more sensitive at detecting deficits in selective attention and response inhibition. However, mean values on all other Stroop test and TMT scores indicated that the longer abstinence duration group performed better in comparison to the shorter abstinence duration group, revealing a trend of lesser cognitive impairment in patients with frequent and longer abstinences. The findings of this study are supported by observations made by a recent study comparing Korsakoff and non-Korsakoff alcoholics on neuropsychological tests of func-tioning of the prefrontal brain.^24^ Periods of abstinence provide time for the recovery of cognitive functions as well as prevent further damage to the frontal lobes—a fact which explains the better executive functions observed in the longer abstinence group.

Regarding the association of alcohol consumption variables and executive functions, neither the duration of dependence nor the amount of intake were found to be associated with performance on the TMT or Stroop test. Our findings do not support the observations made by Kuruoglu *et al.,*^25^ who found significant correlation between the duration of alcohol intake and decreased frontal perfusion in a group of alcohol-dependent patients with a continuous pattern of intake (mean duration of dependence duration 17.4 years). In the present study, the mean duration of dependence was 10.94 years and 8 out of the 30 patients described a pattern with frequent periods of abstinence. The difference in pattern of alcohol intake could account for the observed discord between the two studies. Interestingly, the duration of alcohol use was not related to performance on the neuropsychological test but recent consumption and days of sobriety were associated with non-verbal abstract reasoning ability in a recent study by Zinn *et al.*^9^

In conclusion, our study provides further support for anterior brain deficits in patients dependent on alcohol. It also elucidates the effect of patterns of drinking (with or without frequent periods of abstinence) on cognitive deficits. However, it fails to document any association of duration of alcohol dependence and amount of intake with frontal deficits.

Our study strengthens the anterior brain deficit hypothesis in abstinent alcoholic patients. The fact that the patients were not on any psychotropic drugs at the time of assessment rules out medication-related confounders in our findings. Additionally, by ruling out patients with neurological or medical illnesses the impact of these factors on executive functions was nullified at the outset. Collection of data by a single interviewer ensured reliability of the data.

Some limitations of this study are worth mentioning. We could not do an extensive assessment of frontal lobe functions with a wide array of tests, though this does not undermine our findings. Important lacunae included a lack of consideration for the nutritional status of the patients as well as of neuro-imaging correlates.

Despite these shortcomings, the present study has further supported the previous findings of frontal deficits in alcohol-dependent patients. It also elucidates that some of these functions are more severely impaired in patients with a regular pattern of drinking. This finding is of help in planning for prevention of relapse in these patients. Since neurocognitive functions have been found to moderate the relationship between coping and substance use outcome, such patients may not be ideal candidates for relapse prevention using coping skills' training, especially during the early days of abstinence.^8^

Future research addressing the above limitations as well as with a longitudinal follow-up design may throw some more light on this issue, especially with regard to assessing the longitudinal outcome in terms of time to relapse and mainte-nance of sobriety.
